# Pharmacokinetic Equations Applied to Obtain New Topological Models in the Search of Antibacterial Compounds

**DOI:** 10.3390/ph18060865

**Published:** 2025-06-10

**Authors:** Jose I. Bueso-Bordils, Gerardo M. Antón-Fos, Rafael Martín-Algarra, Pedro A. Alemán-López

**Affiliations:** 1 Pharmacy Department, Universidad Cardenal Herrera-CEU, CEU Universities C/Ramón y Cajal s/n, Alfara del Patriarca, 46115 Valencia, Spain; 2 Department of Chemistry and Biochemistry, Faculty of Pharmacy, Universidad San Pablo-CEU, CEU Universities, Urbanización Montepríncipe, 28003 Madrid, Spain

**Keywords:** antibacterial, computational chemistry, pharmacokinetics, topological indices, drug-like, QSAR

## Abstract

**Background:** QSAR (Quantitative Structure–Activity Relationships) methods have been the basis for the design of new molecules with a certain activity. The great advantage of QSAR methods is that they can predict the pharmacological activity of compounds without the need to obtain or synthesize them previously. Currently, the development of antibiotic resistance by microorganisms is the most important issue in the treatment of infectious diseases. This elevated resistance is associated with expanded morbidity and mortality, as well as an increase in healthcare costs. The development of new molecules with antibacterial activity is therefore urgently needed. **Methods:** By means of molecular topology, we developed discriminant functions (DF1 and DF2) capable of predicting antibacterial activity. When applied to a database with 6373 chemicals, they selected 266 molecules as candidates, from which 41% have this activity, according to the bibliography. Regression equations determining pharmacokinetic properties such as mean residence time (MRT), volume of distribution (V_D_), and clearance (CL) were applied to the selected molecules. **Results:** We have observed that most antibacterial compounds have pharmacokinetic theoretical values in the intervals 20 > MRT > 0, 3 > V_D_ > 0, and 500 > CL > 0. We have applied these intervals to our antibacterial model with the objective of finding new antibacterials with a good pharmacokinetic profile. We show that they are an effective tool for discriminating antibacterial compounds, increasing the bibliographic success rate to 50.8, 59, and 61.5%, respectively. When drug-like filters are applied to these new models, the vast majority (89.9–100%) of the selected molecules present antibacterial activity. **Conclusions:** Considering these results, these new models could avoid the application of drug-likeness filters when searching for new potential antibacterials. All of this proves the usefulness of these mathematical–topological models.

## 1. Introduction

The introduction of antibacterial drugs into clinical practice in the 1940s was one of the most important interventions for its control and increased the life expectancy of the population by several years. Like vaccines, they saved millions of lives, but they also brought about a revolution in medicine, significantly contributing to progress in fields such as solid organ and hematopoietic stem cell transplantation, survival of premature and immunosuppressed infants, and surgery of prosthetic material and vascular catheters, where infections are especially prevalent and important [1].

Since the mid-20th century, the pharmaceutical industry has intensified efforts to discover new antibiotic molecules from different microorganisms, preferably from soil samples or semisynthetic derivatives thereof. A great variety of these compounds belonging to very diverse families (beta-lactams, glycopeptides, macrolides, aminoglycosides, tetracyclines, etc.) were discovered. It was the golden age for these drugs, and it was believed that the battle against infectious diseases had already been won. In fact, infant mortality and morbidity fell considerably. Research was also conducted on the development of synthetic antimicrobials that were used in human and animal therapeutics, such as quinolones obtained from nalidixic acid, another synthetic agent. Quinolones constitute a widely used class of antibiotics due to their pharmacokinetic qualities, their bactericidal action against many pathogens, and their low toxicity [2].

However, soon after the discovery of antibiotics, the ability of bacteria to mutate and generate resistance to them was reported. Despite early warnings about the perils of their overuse, widespread over-prescription of antibiotics and their use in agriculture resulted in a rise in the growing resistance of some bacteria, such as pneumococcus and staphylococcus, to conventional antibiotics [3]. The extreme versatility and adaptability of microorganisms have prevented a decrease in the prevalence of these diseases since the number of antibiotic-resistant bacteria has multiplied exponentially in the last decades [4]. So much so that the World Health Organization (WHO) already considers antibiotic resistance developed by bacteria as one of the main threats to global health and points out that if the emergence of resistance continues at this rate, infections by antibiotic-resistant bacteria will be the leading cause of death worldwide, surpassing deaths caused by cancer, diabetes, and cardiovascular diseases [5].

The alarming increase in these resistances is, without a doubt, one of the biggest current public health problems since these compounds constitute one of the main tools for controlling and treating bacterial infections, both in human and veterinary medicine. This issue spans multiple sectors and presents several complex challenges. Aside from the medical components, it has economic, ecological, sociological, and developmental dimensions. The loss of effectiveness of these drugs would lead us to a silent pandemic that could make the world and the modern medicine built upon it collapse [6].

In the current context, rapid and inexpensive methods are necessary for the immediate expansion of the therapeutic arsenal that we currently possess. Within this framework, the drug repurposing strategy has emerged, which consists of using drugs that are already on the market and that have other therapeutic purposes to treat infections caused by multi-resistant bacteria [7,8,9]. This approach using known drugs is a common practice in the design and development of new drugs, saving time and resources in clinical and preclinical phases since, for example, toxicological and/or pharmacokinetic assays would have already been carried out previously [10].

In this sense, it is worth highlighting the utility of mathematical–topological models for the prediction of antibacterial activity, which allows obtaining new drugs with antibacterial capacity and which are alternative tools, given the reduction in investment by the pharmaceutical industry in this scope. Thus, within these methods, one can find the Quantitative Structure–Activity Relationship (QSAR) methods, which play an important role since they provide useful information for rational drug design and development with minimal cost. The great advantage of these QSAR methods is that they can predict the pharmacological activity of a compound without the need to previously obtain or synthesize it. This has caused a great rise in the number of studies regarding Computational Chemistry and Virtual Combinatorial Chemistry in the last years [11]. Specifically, in our research group, we use molecular topology, which consists of obtaining a series of topological indices (TIs) that are calculated from the structure of a molecule. In this way, we can classify a molecular structure as active or not active against a certain pharmacological activity using pattern recognition techniques such as linear discriminant analysis, multilinear regression, or random decision forests [12,13].

This field offers an alternative and inexpensive method for the rapid identification of new antibacterial agents. Of special interest are antibiotics with optimum pharmacokinetic properties. Besides modeling biological activity endpoints, QSAR methods can also involve modeling the pharmacokinetic properties of drug candidates prior to their synthesis, ensuring the efficacy of the designed compounds inside biological systems [14]. The inclusion of the prediction of pharmacokinetic and/or pharmacodynamic properties, such as the ability to cross the blood–brain barrier, prediction of cytochrome P450-mediated metabolism, albumin binding, or water solubility, attempt to simultaneously optimize potency and pharmacokinetics [15].

We therefore reiterate QSAR methods as an effective tool to perform rapid virtual screening for potential antibacterials, allowing the prediction of other pharmacokinetic and toxicological properties directly related to activity to find safer and more effective drugs. Thus, one promising possibility appears to be the combination of multiple QSAR models that will generate the most suitable antibacterial compounds, considering not only their activity but also their properties. We want to apply pharmacokinetic prediction equations to generate new antibacterial models with the objective of finding antibacterials with good pharmacokinetic profiles. Furthermore, by introducing pharmacokinetic values, these models select drug-like molecules. Also, the clear definition of a model’s applicability domain (AD) is crucial since it enhances confidence in the predictions and enables the practical application of QSAR models [16]. Indeed, the Organization for Economic Cooperation and Development (OECD) requires assessing the AD of QSAR models as a prerequisite for using QSAR in regulatory contexts [17].

As pharmaceutical companies increasingly step back from antibiotic research—largely due to high costs and limited financial incentives—there is an urgent need for smarter, more affordable ways to discover new treatments. This is where QSAR modeling comes in. Unlike traditional methods that require physically creating and testing compounds, QSAR can predict a molecule’s potential just by analyzing its structure. It is a practical and efficient alternative to high-throughput screening, which demands extensive lab resources, or AI and machine learning approaches that often need massive, well-curated datasets. QSAR strikes a balance: it is accessible, cost-effective, and scientifically robust. Especially when combined with molecular topology, it becomes a powerful tool for quickly identifying promising antibacterial candidates—something we desperately need in the fight against resistant bacteria.

## 2. Results and Discussion

After extending thorough bibliographic research on antibacterial activity from a previous study [18], a total of 266 molecules from our dataset were selected by the AB model as potential antibacterials. Of these, 109 have proven antibacterial activity bibliographically. That is, considering only the scientific literature, the AB model correctly classified as antibacterial in 41% of the selected compounds.

To build the discriminant functions, we used four groups: two training groups with 38 active and 38 inactive compounds and two test groups with 12 active and 11 inactive compounds, which allowed for assessing the quality of the selected discriminant functions.

The chosen functions were as follows:

DF1 = −27.13819 − 4.7164*S_=C<_* + 1.27264*S_-NH-_* − 0.30296*S_=O_* + 43.39831^0^*C* − 13.57036^3^*C_c_*                 N = 76     λ = 0.2653522     F = 38.76(1)

DF2 = −3.27384 − 3.14402*S_=C<_* + 0.66311*S_-NH_*_2_ + 1.44735*S_-NH-_* + 1.15103*S_=N-_* − 0.47139*S_-OH_* − 4.41362*S_aSa_*                   N = 76     λ = 0.3138095     F = 25.146(2)

where the quality of the discriminant functions is evaluated by the Wilks’ λ parameter (also known as U-statistic), which is obtained by a multivariate analysis of variance statistics that tests the equality of group mean values for the variables in the DFs.

We plotted the corresponding PDDs for every function to visualize the values of the function in which the probability of classifying a compound as active or inactive is maximum. In other words, to find areas where the overlap between the two groups of molecules is minimal. The maximum expectancy zone for new antibacterials is the range of 0–12 for DF1 and the range of 2–12 for DF2 in both the training and the test sets [18].

With the purpose of finding three mathematical models able to identify antibacterials with good pharmacokinetic profiles, RFs built based on the bibliographic information of a limited number of antibacterial quinolones were applied to the same chemical graphs used for DF1 and DF2, only that this time there were two, not four, large sets of molecules: one with 50 antibacterial quinolones and another one comprising 49 inactive quinolones and quinolone-related structures.

We aimed to build three models by combining DF1 and DF2 with a pharmacokinetic equation, which related, respectively, the mean residence time (MRT) in hours, the volume of distribution (V_D_) in L/kg, and the clearance (CL) in mL/min [19,20]:

MRT = −79.3319 − 4534.37^10^*χ^v^_ch_* + 13.6939^2^*κ_α_* − 2.21175*S_-O-_*     N = 14       r^2^ = 0.85378       r^2^_cv_ = 0.74026       SEE = 6.306649       F = 19.46 (3)

V_D_ = 10.1212 + 0.704187*S_-NH2_* − 0.612674*S_-Cl_* − 0.615152*G*_2_     N = 16       r^2^ = 0.85816       r^2^_cv_ = 0.75868       SEE = 0.694465       F = 24.20 (4)

CL = 8981.08 + 1596.68^6^*χ_ch_* − 109.481*S_>N-_ −* 2637.68*NI*_2_     N = 12       r^2^ = 0.92043       r^2^_cv_ = 0.70282       SEE = 65.42953       F = 30.85 (5)

where r^2^_cv_ is obtained by cross-validation analysis using the leave-one-out method [21].

Since the equations have been obtained from antibacterial quinolones, it is expected that the molecules selected by the models maintain the same mechanism of action (inhibition of DNA gyrase and/or topoisomerase IV) and similar pharmacokinetic properties (MRT, V_D,_ or CL).

**Figure 1:**
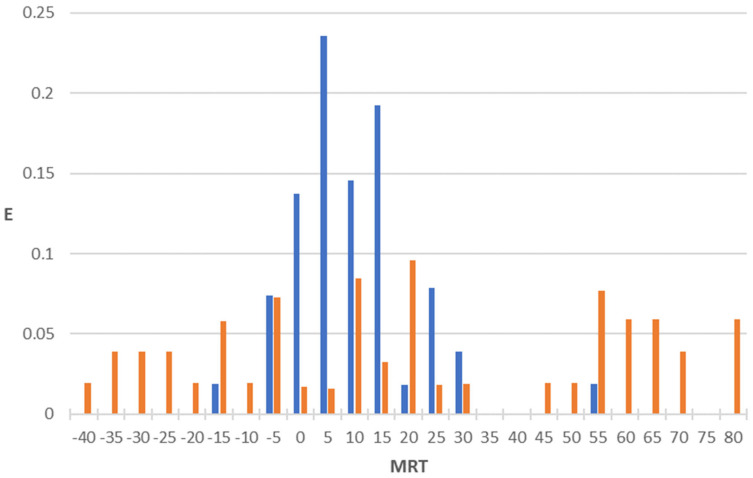
PDD for mean residence time (hours) property as obtained by using the regression function MRT: blue bars, antibacterial compounds; orange bars, non-antibacterial compounds. Highest property expectancy (E): 0 < MRT < 20.

**Figure 2:**
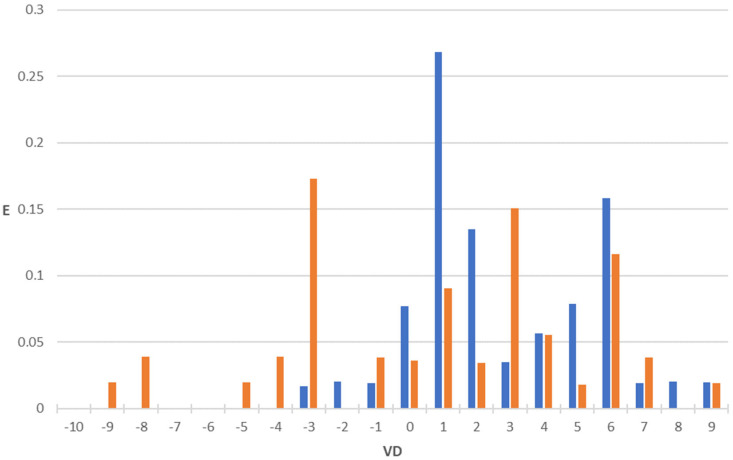
PDD for volume of distribution (L/kg) property as obtained by using the regression function V_D_: blue bars, antibacterial compounds; orange bars, non-antibacterial compounds. Highest property expectancy (E): 0 < V_D_ < 3.

**Figure 3:**
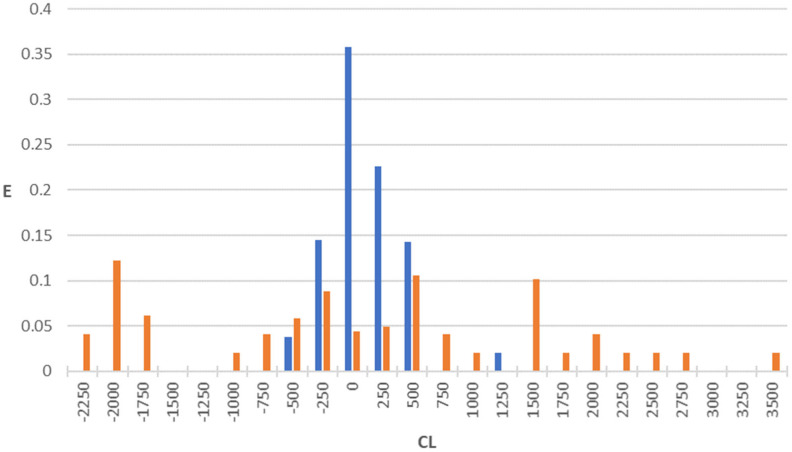
PDD for clearance (mL/min) property as obtained by using the regression function CL: blue bars, antibacterial compounds; orange bars, non-antibacterial compounds. Highest property expectancy (E): 0 < CL < 500.

After the selection of the three equations constituting the pharmacokinetic property in the topological model, we built the corresponding PDDs. Application of these equations to the two large sets of drugs (one comprising compounds exhibiting antibacterial activity and the other not possessing it) reveals that most of the actives are gathered within a specific interval, while the inactives are generally scattered or fall outside this interval. The PDDs were obtained by using Equations (3)–(5) (MRT, V_D,_ and CL, respectively) along with the highest property intervals for each function, which are shown in Figure 1, Figure 2 and Figure 3. Thus, the intervals derived from these PDDs establish the AD for each model.

The derived discriminant functions are applicable not only to novel quinolone derivatives outside the scope of the original dataset but also to structurally unrelated compounds, provided they exhibit analogous mathematical–topological descriptors. The robustness of the topological approach has been substantiated through its successful implementation across a broad spectrum of pharmacological classes [13].

We observe that some calculated values are negative (Figure 1, Figure 2 and Figure 3). The explanation for this fact is that the function assigns these negative values to very small values of the property. Such is the case of antibacterial quinolones with observed pharmacokinetic values within the first positive interval (MRT: 0–5 h; VD: 0–1 L/kg; CL: 0–250 mL/min), whose calculated pharmacokinetic values are negative.

### 2.1. Antibacterial + Mean Residence Time Model (AB+MRT Model)

Considering the obtained results, an optimized topological MRT model for antibacterial activity can be formulated through DF1, DF2, and MRT (see Equations (1)–(3) and Figure 1), where DF1 and DF2 address antibacterial activity and MRT addresses mean residence time property. A molecule would be classified as active if it fulfilled the following requirements:

12 > DF1 > 0

12 > DF2 > 2

20 > MRT > 0

After applying all three functions (Equations (1)–(3)) to our dataset, we identified 59 compounds as theoretically active because their DF1, DF2, and MRT values (AB+MRT model) fell within the predefined intervals. Of these, 30 compounds (50.8%) had demonstrated antibacterial activity, according to the bibliography (see Supplementary Section S2).

Figure 1 indicates that the vast majority of antibacterials fall within the MRT equation’s selected interval of calculated values (74% of the antibacterials in the 20 > MRT > 0 interval), while non-antibacterial compounds’ calculated values are mostly dispersed (only 18.4% in the same interval). It must be noted that the scientific literature generally refers to half-life (t_1/2_) or beta-phase half-life (bpt_1/2_) rather than MRT, where MRT = t_1/2_/ln2 in the case of one-compartment models and MRT = bpt_1/2_/ln2 in the case of two-compartment models [22]. This consistency MRT experimental values, where first- and second-generation quinolones such as enoxacin, norfloxacin or ciprofloxacin with low to medium elimination rate (2 h ≤ t_1/2_ ≤ 4 h or 2.89 h ≤ MRT ≤ 5.77 h) while later quinolones such as gatifloxacin, moxifloxacin, or trovafloxacin, whose structures’ optimization usually led to longer elimination rates (t_1/2_ ≤ 13 h or MRT ≤ 18.76 h) [23,24,25,26,27,28,29,30,31,32], supports the validity of this model.

The encouraging performance of the AB+MRT model suggests that integrating pharmacokinetic parameters could refine our screening process. With this in mind, we next turn our attention to the volume of distribution.

### 2.2. Antibacterial + Volume of Distribution Model (AB+VD Model)

The volume of distribution (V_D_), which is the volume of body fluid into which a drug’s dose is dissolved, is an important determinant of drug concentration. An optimized topological V_D_ model for antibacterial activity can be formulated through DF1, DF2, and V_D_ (see Equations (1), (2), and (4) and Figure 2), where DF1 and DF2 address antibacterial activity and V_D_ provides additional insight into the compounds’ volume of distribution. A molecule would be classified as active if it fulfilled the following requirements:

12 > DF1 > 0

12 > DF2 > 2

3 > V_D_ > 0

By applying the proposed topological model (AB+V_D_ model) to our dataset, 39 compounds were selected as theoretically active, of which 22 (59%) were accurately classified as active according to the bibliography (see Supplementary Section S2).

In the case of quinolones, their V_D_ is generally greater than that of total body water, which suggests a wide distribution of these compounds throughout the body due to sequestration in the intracellular fluid of certain tissues due to their moderately lipophilic nature [22,33,34]. However, key structural modifications to improve the pharmacokinetics of third- and fourth-generation quinolones, such as the addition of an amino or methyl group in R_5_ (e.g., sparfloxacin and grepafloxacin, respectively) or the presence of piperazine or azabiclylic ring in R_7_ (e.g., gatifloxacin and garenoxacin, respectively) increased the quinolones’ lipophilicity and, thus, their experimental values of V_D_, resulting in a high variability among the group [24,25,26,27,28,29,30,31,32,35,36].

Figure 2 indicates that antibacterials tend to cluster within the V_D_ equation’s selected interval of calculated values (52% of antibacterials in the 3 > V_D_ > 0 interval), while non-antibacterial compounds’ calculated values are more scattered (only 20.4% fall in this interval). Therefore, these results align well with known distribution patterns observed in quinolones, where modifications such as the addition of amino or methyl groups have led to significant variations in V_D_.

Building upon the insights gained from the AB+MRT model, the AB+V_D_ model adds another pharmacokinetic characterization. In our continuous effort to enhance predictive accuracy, we now extend our approach to include clearance.

### 2.3. Antibacterial + Clearance Model (AB+CL Model)

Clearance (CL) is a key parameter that indicates the volume of body fluid from which the drug is completely removed per unit time and is critical for understanding a drug’s elimination and duration of action. An optimized topological CL model for antibacterial activity can be formulated by combining DF1, DF2, and CL (see Equations (1), (2), and (5) and Figure 3), where DF1 and DF2 assess antibacterial activity while CL further refines the profile based on elimination characteristics. A molecule would be classified as active if it fulfilled the following requirements:

12 > DF1 > 0

12 > DF2 > 2

500 > CL > 0

When applying the AB+CL model to our dataset, 39 compounds were selected as potential antibacterials, of which 24 (61.5%) were confirmed as active, according to the bibliography (see Supplementary Section S2).

Regarding clearance (CL), changes in quinolones’ elimination were observed over time: early quinolones (e.g., enoxacin, levofloxacin and lomefloxacin) were eliminated primarily by renal clearance, with high observed CL values, whereas later quinolones (e.g., grepafloxacin, moxifloxacin and trovafloxacin) were modified to be more lipophilic, and hence eliminated primarily by hepatic clearance, which tended to show lower observed CL values [2,24,25,26,27,28,29,30,31,32].

Figure 3 illustrates that antibacterial compounds’ calculated values are strongly clustered in the CL equation’s selected interval (62% of antibacterials in the 500 > CL > 0 interval), in contrast to non-antibacterial agents, of which only 12.2% fall within this range. Thus, these findings are consistent with the changes observed over time in quinolone clearance, where shifting from primarily renal to hepatic elimination results in differing CL values.

Hence, by incorporating additional pharmacokinetic parameters, our antibacterial activity models refine the selection of compounds with optimal pharmacokinetic profiles. This approach not only minimizes false positives but also enhances the overall drug-likeness of the selected candidates.

### 2.4. Study of Drug-like Properties in Compounds Selected by Models

To demonstrate in vivo activity of a substance, in addition to having the correct stereoelectronic properties to bind with the receptor, it must be safe and have the appropriate pharmacokinetic properties to reach it. This means that most of the designed compounds do not successfully succeed in phase I clinical trials [37].

The search for novel antibacterial agents is carried out based on a two DF classification model and their PDDs applied to antibacterials and non-antibacterials achieved in previous work [38]. After the application of such a model (as described under Materials and Methods) to a large dataset comprising 6373 chemicals (including either drugs or chemical reactants), those 266 molecules passing the model requirements were selected (4.2% of the total) of which, considering only the bibliographic data, the AB model correctly classified as antibacterial at least 41% of the selected compounds.

Later, the same group of antibacterials and non-antibacterials is screened for three different pharmacokinetic properties (functions MRT, V_D,_ and CL and their respective PDDs [19,20]), which leads to 3 different models to search for potential antibacterial with optimum pharmacokinetic properties.

When three functions along with their PDDs are used, the accuracy for active compounds decreases, whereas that for inactive ones increases, so the probability of a false active compound being selected decreases. In these cases, the proportion of correctly classified active compounds rose to 50.8, 59, and 61.5% (AB+TRM, AB+V_D,_ and AB+CL, respectively). This suggests that the values obtained from Equations (3)–(5) are related to TIs that are somehow measures of antibacterial activity, so these equations can also be used as discriminant functions for this type of activity.

Since Lipinski’s rules, many drug-like (DL) properties have been developed to predict the appropriate pharmacokinetic behavior of substances. Thus, an extra evaluation of drug-likeness by application of constitutional indices (number of atoms, nAT [39]), molecular properties (Ghose–Crippen molar refractivity, AMR [40]; topological polar surface area, TPSA [41]; rotatable bond number, RBN [42]) and DL indices (Lipinski’s rule of five, Ro5 [43]), key for oral absorption, is performed using alvaMolecule [38] to all discussed models, including the previously published antibacterial model [18] (see Supplementary Section S3). Table 1 summarizes the percentage of correct classification of the different models obtained for the DL filters selected (Table 1).

As can be seen in Table 1, all the pharmacokinetic equations increased the percentage of molecules with DL properties with respect to the initial database and the molecules selected by the AB model, which increases the probability of finding active molecules with optimum pharmacokinetic properties.

While each of the three models—AB+MRT, AB+V_D_, and AB+CL—demonstrated improved predictive power over the base AB model, their performance varied in terms of accuracy and drug-likeness enrichment. The AB+CL model achieved the highest bibliographic success rate, correctly identifying 61.5% of compounds as antibacterial, followed closely by AB+V_D_ (59%) and AB+MRT (50.8%). In terms of drug-likeness, all three models significantly outperformed the original dataset, with the AB+MRT and AB+CL models achieving 100% compliance with key filters such as Lipinski’s Ro5 and molar refractivity. These findings suggest that while all models are effective, the AB+CL model offers the best overall balance between predictive accuracy and pharmacokinetic relevance, making it a particularly promising tool for the virtual screening of antibacterial candidates.

Finally, the compounds showing a previously known antibacterial activity according to an exhaustive literature search were taken out of the pool of selected candidates so that we finally obtained a group of novel and (potentially) active candidates. We believe that the remaining compounds that, up to our knowledge, have not been previously described as antibacterials (see Supplementary Section S4) could be strong candidates to increase the currently available antimicrobial arsenal. We think that only these data justify and demonstrate the great utility of these new models.

## 3. Materials and Methods

### 3.1. Compound Selection

A mathematical–topological antibacterial prediction model (AB model) previously described by our research group [18] is used to find new antibacterial compounds from a database retrieved from the Index Merck (IM) [44], which gathers 10250 marketed compounds, including pharmaceutical drugs, biological products, and chemical reactants. A series of modifications explained below had to be carried out to transform them into chemical graphs that our index calculation software programs, DESMOL13 [45] and MOLCONN-Z [46], could use. The molecular descriptors used were computed from the adjacency topological matrix obtained from the hydrogen-depleted graphs using the drawing program ChemDraw 12.0 Professional from the ChemOffice 2010 software package (these descriptors are described in Supplementary Section S1 along with their definitions and references).

Firstly, stereochemistry is removed from all molecules since the software only supports 2D graphs. This had to be performed one molecule at a time, changing the bonds that indicated chirality for flat bonds. Furthermore, this software does not accept molecules with more than 99 bonds from end to end of the molecule. Thus, molecular entities exceeding 99 bonds had to be removed. Moreover, we were only able to calculate the indexes of molecules containing C, F, Cl, Br, I, O, N, S, P, B, Si, Ge, Sn, Pb, and H. Therefore, each molecule had to be analyzed to detect any other atoms and delete them. Moreover, an automatized data curation of the molecular structures (duplicate analysis, removal of undesired characteristics such as mixture, salts, polymers, organometallic, and inorganic substances, fixing erroneous molecular representation, and checking for anomalies) is performed using alvaMolecule 2.0.6 [38]. The curation of molecular data can be one of the most time-consuming phases of the model-building process; it often requires human expertise to check molecular structures, even manually, to identify potential problems, and it certainly is a mandatory task when working with software tools that are not able to calculate molecular descriptors for such types of chemical structures. Having taken all these considerations, we were able to build our own dataset with 6373 compounds of approved and experimental drugs (62.2% of the IM database), whose chemical graphs were drawn and saved in “mol” format.

### 3.2. Linear Discriminant Analysis and Multilinear Regression

Linear Discriminant Analysis (LDA) and Multiple Linear Regression (MLR) are commonly employed techniques in the development of QSAR models. However, a significant limitation of these methods is their inability to handle variables with discrete values, as such data cannot be effectively analyzed using these statistical approaches since this type of index would lead to overfitting [47].

The aim of LDA, a heuristic algorithm capable of distinguishing between two or more categories of entities, is to find linear functions that allow one to discriminate between those categories. In one of our previous studies [18], two large sets comprising quinolones with proven antibacterial activity and quinolones and quinolone-related structures without such activity, respectively, were considered. Quinolones represent a well-characterized and diverse class of compounds, offering a rich dataset that enhances the accuracy and reliability of the predictive equations [47].

Chemical graphs used to calculate the AB model formed by two discriminant functions (DF1 and DF2) were randomly split into training (molecules used to build the DFs) and test (molecules used to ensure the quality of the DFs) groups for each set using the statistical package called BMDP module 7D [48]. The discriminant ability of antibacterial activity is assessed in terms of the proportion of correct classification in each group, and a search of potential antibacterial compounds is performed in a big dataset with a vast majority of non-related structures [18], which identified substances with antibacterial activity previously described in the bibliography (41% of the selected compounds). Moreover, we also demonstrated that it is possible to obtain a high degree of molecular characterization of certain pharmacokinetic properties (mean residence time after oral administration, MRT; volume of distribution, V_D_; clearance, CL) by an adequate choice of TIs in the form of regression functions (RF) [19,20] using MLR, performed by a BMDP 7.0 software module 9R [48].

Experimental procedures used to obtain property values were carefully reviewed. Specifically, activities against various microorganisms expressed as different MIC values—derived from in vitro assays conducted in accordance with CLSI guidelines [49]. Additionally, data obtained from studies involving healthy human subjects were used to determine pharmacokinetic properties.

Randomness tests were conducted to detect potential spurious correlations in the regression models. This involved randomly permuting the property values of each compound and performing linear regression with the selected independent variables. The outcomes of these tests were visualized by plotting the correlation coefficient (r^2^) against the cross-validated prediction coefficient (r^2^_cv_), providing a graphical assessment of model robustness [21]. It is also recommended that the number of indices chosen is well below the number of molecules used to build the functions to avoid possible fortuitous correlations so that the optimal relationship between them is the one that achieves a better prediction with the minimum number of descriptors possible, in order to avoid random variation as much as possible [50].

### 3.3. Pharmacological Distribution Diagrams

In recent work [19,20], we found that connectivity functions can be used not only to predict properties or classify chemical structures according to activity classification but also to predict the activity of a given compound in a specific pharmacological context. This is accomplished by using pharmacological distribution diagrams (PDD), which allow us to estimate the probability of a given molecule being active. These diagrams have been proven useful with various therapeutic categories (e.g., antihistamines, bronchodilators, and anti-tubercular drugs [51,52,53]).

After selecting the DFs and RFs, we built the corresponding pharmacological distribution diagrams (PDD). These plots are useful to determine the intervals of the discriminant function in which the expectancy, E, to find active molecules is maximum. PDDs are histogram-like plots of connectivity functions in which the expectancies appear on the ordinate axis [54]. For an arbitrary interval of values of a given function, we can define the expectancy of activity as E_a_ = a/(i + 1), where “a” is the number of active compounds in the interval divided by the total number of active compounds, and “i” is the number of inactive compounds. The expectancy of inactivity is defined in a symmetrical way, as E_i_ = i/(a + 1). This representation provides good visualization of the regions of minimum overlap and selects regions in which the probability of finding improved molecules is maximum.

### 3.4. Topological Models

By means of MLR and LDA techniques, three topological mathematical models comprising three functions per model were achieved for antibacterial activity. The different topological models were formed by combining both DFs (AB model) with an RF. Furthermore, PDDs allowed us to carry out the assignment of thresholds useful to discriminate active from inactive compounds with the highest probability of success. Only the molecules predicted as active by all three functions comprised in each of the models within the predetermined thresholds were identified as potential antibacterial drugs with an adequate pharmacokinetic profile. This way, the number of false actives will be minimized.

## 4. Conclusions

Currently, the emergence of antibiotic resistance in microorganisms is one of the biggest issues that have appeared in recent years in the treatment of infectious diseases. MT has proven to be a powerful tool in the design and development of new antibacterials, considering the increasing appearance of multi-resistant bacteria and the lack of investment from the pharmaceutical industry. Furthermore, RFs aiming at predicting pharmacokinetic properties have been proven to have the ability to group active compounds effectively, which suggests a close relationship between them and the antibacterial activity of compounds. By combining them with DFs, we developed three QSAR models with clear-cut intervals in which the probability of finding active molecules reaches its maximum according to the corresponding PDDs.

In this study, we applied these three models to a dataset derived from the IM database, identifying molecules with antibacterial activity previously described in the bibliography with outstanding discriminant ability according to the success rates. The model has also selected compounds whose antibacterial activity, up to our knowledge, has not been proven yet, which could be interesting candidates to be studied, providing new possible action mechanisms and thus increasing contribution to tackle antibiotic resistance.

These models provide useful information for a better understanding of how the molecular structure may affect the antibacterial activity of a molecule. With this information, it is simpler to understand how topological characterization of chemical structures may affect the antibacterial activity of a compound and therefore optimize existing drugs, which is a cost-effective approach to obtain new treatments against resistant bacteria.

We can conclude that the results obtained in this study confirm QSAR and molecular topology as extremely useful and cost- and time-effective tools, where DL filters could be avoided in the virtual screening of new antibacterials. The new models generated could be of application in drug repurposing, where we find thousands of safe and efficient candidates to be tested for new pharmacological activities.

Thus, having demonstrated again the efficacy of this predictive tool, our present and future work is focused towards their validation by means of experimental in vitro testing.

## Figures and Tables

**Table 1:** Percentage of molecules from the starting dataset and different models that fulfill the drug-like filters.

DL Filter ^a^	IM Dataset ^b^	AB Model ^c^	AB+MRT Model ^d^	AB+V_D_ Model ^e^	AB+CL Model ^f^
Ro5 ≥ 0.75	75.8	91.6	100	100	100
20 ≤ nAT ≤ 70	77.5	91.6	100	97.4	100
40 < AMR < 130	82.6	93.4	100	94.6	100
TPSA < 140	78.9	82.8	89.9	97.4	94.9
1 ≤ RBN ≤ 9	81.7	85.3	100	92.1	94.9

^a^ Ro5: Lipinski’s rule of five; nAT: number of atoms; AMR: molar refractivity; TPSA: topological polar surface area; RBN: rotatable bond number. ^b^ Percentage of molecules from the IM dataset that fulfill each of the DL filters. ^c^ Percentage of molecules from the IM dataset that are theoretically active according to the AB model and fulfill each of the DL filters. ^d^ Percentage of molecules from the IM dataset that are theoretically active according to the AB+MRT model and fulfill each of the DL filters. ^e^ Percentage of molecules from the IM dataset that are theoretically active according to the AB+V_D_ model and fulfill each of the DL filters. ^f^ Percentage of molecules from the IM dataset that are theoretically active according to the AB+MRT model and fulfill each of the DL filters.

## Data Availability

Data is contained in the paper.

## Supplementary Materials

The following supporting information can be downloaded at https://www.mdpi.com/article/10.3390/ph18060865/s1. Supplementary Section S1: Symbols and Definitions of Topological Indices used with DESMOL13 and MOLCONN-Z programs; Supplementary Section S2: Compounds selected by the models as candidates with proven antibacterial activity; Supplementary Section S3: SMILES file with the list of molecules and properties calculated by alvaMolecule; Supplementary Section S4: Compounds selected by the models as candidates without proven antibacterial activity. References [55,56,57,58,59,60,61,62,63,64,65,66,67,68,69,70,71,72,73,74,75,76,77,78] have been cited to Supplementary Materials.
