# Supplementary material for: Pharmacokinetic Equations Applied to Obtain New Topological Models in the Search of Antibacterial Compounds
Source: Pharmaceuticals (Basel). 2025 Jun 10;18(6):865. doi: 10.3390/ph18060865 (PMC12195795; doi:10.3390/ph18060865)
Supplement: Supplementary file 1 [file pharmaceuticals-18-00865-s001.zip › Suppl Section S1.pdf]

Suppl. Section S1: Symbols and Definitions of Topological Indices used with DESMOL13[a] and MOLCONN-Z[b] programs.

Symbol	Name	Definition	Ref.
Connectivity Indices			
${}^{k}\chi_{t}$<br><br>k=0-10<br>t=p,c,pc	Kier-Hall indices of order k and type path (p), cluster (c) and path-cluster (pc)	$^{k}\chi_{t} = \sum_{j=1}^{^{k}n_{t}} \left(\prod_{i \in S_{j}} \delta_{i} \right)^{-1/2}$<br>$\delta_i$: number of bonds, σ o π, of the atom i to non-hydrogen atoms.<br>S_j,: jth substructure of order k and type t.	c
${}^{k}\chi_{t}^{v}$<br><br>k=0-10<br>t=p,c,pc	Kier-Hall indices of order k and type path (p), cluster (c) and path-cluster (pc)	$^{k}\chi_{t}^{v} = \sum_{j=1}^{^{k}n_{t}} \left(\prod_{i \in S_{j}} \delta_{i}^{v} \right)^{-1/2}$<br>$\delta_i^v$: Kier-Hall valence of the atom i.<br>S_j,: jth substructure of order k and type t.	b
${}^{k}D_{t}$<br>k=0-10<br>t=p,c,pc	Connectivity differences of order k and type path (p), cluster (c) and path-cluster (pc)	$^{k}D_{t} = {}^{k}\chi_{t} - {}^{k}\chi_{t}^{v}$	b
${}^{k}C_{t}$<br>k=0-10<br>t=p,c,pc	Connectivity ratios of order k and type path (p), cluster (c) and path-cluster (pc)	$^{k}C_{t} = {}^{k}\chi_{t} / {}^{k}\chi_{t}^{v}$	b
Charge Indices			
G_k<br><br>k=1-5	Topological charge indices of order k	$G_{k} = \sum_{i=1}^{N-1} \sum_{j=i+1}^{N} \left\| \mathbf{M}_{ij} - \mathbf{M}_{ji} \right\| \delta(k, \mathbf{D}_{ij})$<br>**M**=**A.Q**: product of the adjacency and inverse squared distance matrices for the hydrogen-depleted molecular graph.<br>**D**: distance matrix.<br>δ: Kronecker delta.	d
G_k^v<br><br>k=1-5	Valence topological charge indices of order k	$G_{k}^{v} = \sum_{i=1}^{N-1} \sum_{j=i+1}^{N} \left\| \mathbf{M}_{ij}^{v} - \mathbf{M}_{ji}^{v} \right\| \delta(k, \mathbf{D}_{ij})$<br>$\mathbf{M}^v$=$\mathbf{A}^v$.**Q**: product of the electronegativity-modified adjacency and inverse squared distance matrices for the hydrogen-depleted molecular graph.<br>**D**: distance matrix.<br>δ: Kronecker delta.	d
J_k, J_k^v<br>k=1-5	Pondered topological charge indices of order k	$J_{k} = \frac{G_{k}}{N-1}$ $\quad J_{k}^{v} = \frac{G_{k}^{v}}{N-1}$	d
Electrotopological Indices			
S(i)	Sum of electrotopological indices for a type of atom i	$S_{i} = I_{i} + \Delta I_{i}$<br>$I_i$: intrinsic state value of atom i.<br>ΔI_i: perturbation of I_j on I_i with the form as $\Delta I_{ij} = (I_i - I_j)/D^2_{ij}$	e
Gmaxpos	Gmaxpos index	Maximum positive value for the	f

		electrotopological state of non-hydrogen atoms in the molecule.	
Hmaxpos	Hmaxpos index	Maximum positive value for the electrotopological state of hydrogen atoms in the molecule.	c
Molecular Properties			
N	Molecular size	Number of non-hydrogen atoms.	g
L	Length	Maximum distance between atoms in terms of bonds.	h
PR_i	PR0 a PR3	Number of pairs of ramifications separated by *i* atoms.	f
R	Ramification	Number of simple structural branches.	f
V_k k=3,4	Vertexes of grade *k*	Number of atoms with *k* bonds, σ or π, with other atoms (hydrogens not included).	f
knotp	knotp index	Difference between ${}^3\chi_c$ and ${}^4\chi_{pc}$ indices	i
knotpv	knotpv index	Difference between ${}^3\chi^v{}_c$ and ${}^4\chi^v{}_{pc}$ indices	h
numhbd	numhbd index	Number of hydrogen-donating atoms in the molecule.	h
numhba	numhbd index	Number of hydrogen-accepting atoms in the molecule.	h
Information Indices			
$I_{Shannon}$	Shannon index	Index based on the atomic diversity of the molecule.	g
NI, NI2	NI, NI2 indices	Indices based on information theory.	g
BonIdW	Bonchev IdW(G) index	Index based in the distribution of topological distances in the molecule.	j
BonIdG	Bonchev Id(G) index	Index based on the number of order 2 subgraphs in the molecule.	i
Molecular Shape Indices			
${}^n\kappa$ n=1-3	Kappa index of order *n*	${}^1\kappa = N(N-1)^2/({}^1P)^2$ ${}^2\kappa = (N-1)(N-2)^2/({}^2P)^2$ ${}^3\kappa = (N-3)(N-2)^2/({}^3P)^2$ [N even; N>3] ${}^3\kappa = (N-1)(N-3)^2/({}^3P)^2$ [N odd; N>3] N: number of non-hydrogen atoms. ${}^{1,2,3}P$: number of type path subgraphs of order 1, 2, 3.	k
${}^n\kappa_\alpha$ n=1-3	Kappa-alpha index of order *n*	${}^1\kappa = N+\alpha(N+\alpha-1)^2/({}^1P+\alpha)^2$ ${}^2\kappa = (N+\alpha-1)(N+\alpha-2)^2/({}^2P+\alpha)^2$ ${}^3\kappa = (N+\alpha-3)(N+\alpha-2)^2/({}^3P+\alpha)^2$ [N even; N>3] ${}^3\kappa = (N+\alpha-1)(N+\alpha-3)^2/({}^3P+\alpha)^2$ [N odd; N>3] N: number of non-hydrogen atoms. ${}^{1,2,3}P$: number of type path subgraphs of order 1, 2, 3. $\alpha = \Sigma[(R_i / R_{Csp3})-1]$ R_i: covalent radius for atom *i*.	l

		R_{Csp3}: covalent radius for atom *Csp*^{3}.	
Φ	Phia flexibility index	$\Phi = ({}^1\kappa_\alpha \cdot {}^2\kappa_\alpha) / N$<br>N: number of non-hydrogen atoms.	m
Global Topological Indices			
Sum-I	Sum of the intrinsic state values	$I_i = \frac{\delta_i^v + 1}{\delta_i}$; $Sum - I = \sum I_i$<br>δ_i, number of bonds, σ o π, of the atom *i* to non-hydrogen atoms.<br>δ_i^v, Kier-Hall valence of the atom *i*.	e
Sum-ΔI	Sum of the change in intrinsic state values	$\Delta I = \frac{I_i - I_j}{r_{ij}^2}$; $Sum - \Delta I = \sum \frac{I_i - I_j}{r_{ij}^2}$<br>$r_{ij}$, number of vertexes between *i* and *j* atoms.	e
TETS2	Total electrotopological state index	Sum of all the electrotopological indices in the molecule.	e
TTd4	TTd4 index	Sum of the intrinsic state of all the atoms in the molecule.	e
nclass	Nclass index	Maximum number of topological vertices of a graph by the shortest path	n
W	Wiener index	Sum of the topological distances between all the non-hydrogen atoms by the shortest path.	o
Wp	Wiener polarity index	Number of pairs of atoms with a distance of 3 bonds.	p
Ww	Hyper-Wiener index	Sum of the topological distances and the squared topological distances between all the non-hydrogen atoms by the shortest path.	q
Wt	Total Wiener index	Sum of the topological distances between all the non-hydrogen atoms by the longest path.	r
PlattF	PlattF index	Sum of the grades of the bonds.	s

a. DESMOL13 software; Unidad de Investigación de Diseño de Fármacos y Conectividad Molecular, Facultad de Farmacia, Universitat de València: Valencia, **2000**.
b. Hall, L. H. MOLCONN-Z software; Eastern Nazarene College: Quincy (Massachusetts), **1995**.
c. Kier, L.B.; Hall, L.H. General definition of valence delta-values for molecular connectivity. *J. Pharm. Sci.*, **1983**, *72*(10), 1170-1173.
d. Gálvez, J.; García-Domenech, R.; Salabert, M.T.; Soler, R. Charge indexes. New topological descriptors. *J. Chem. Inf. Comput. Sci.*, **1994**, *34(3)*, 520-525.
e. Kier, L.B.; Hall, L.H. The E-state as an extended free valence. *J. Chem. Inf. Comput. Sci.*, **1997**, *37*(3), 548-552.
f. Basak, S.C.; Mills, D. Quantitative structure-property relationships (QSPRs) for the estimation of vapor pressure: a hierarchical approach using mathematical structural descriptors. *J. Chem. Inf. Comput. Sci.*, **2001**, *41*(3), 692-701.
g. Shannon, C.E.; Weaver, W. *The Mathematical Theory of Communication*; University of Illinois Press: Urbana, **1949**.

h. Moliner, R.; Garcia, F.; Galvez, J.; Garcia-Domenech, R.; Serrano, C. Nuevos índices topológicos en conectividad molecular. Su aplicación a algunas propiedades fisicoquímicas de un grupo de hidrocarburos alifáticos. *An. Real Acad. Farm.*, **1991**, *57*, 287-298.
i. Cummins, D.J.; Andrews, C.W.; Bentley, J.A.; Cory, M. Molecular diversity in chemical databases: comparison of medicinal chemistry knowledge bases and databases of commercially available compounds. *J. Chem. Inf. Comput. Sci.*, **1996**, *36*(4), 750-763.
j. Bonchev, D.; Trinajstic, N. On topological characterization of molecular branching. *Int. J. Quantum Chem.*, **1978**, *14*(S12), 293-303.
k. Kier, L.B. A shape index from molecular graphs. *Quant. Struct.-Act. Relat.*, **1985**, *4*(3), 109-116.
l. Kier, L.B. Distinguishing atom differences in a molecular graph shape index. *Quant. Struct.-Act. Relat.*, **1986**, *5*(1), 7-12.
m. Kier, L.B. An index of molecular flexibility from Kappa shape attributes. *Quant. Struct.-Act. Relat.*, **1989**, *8*(3), 221-224.
n. Suay-García, B.; Alemán-López, P.; Bueso-Bordils, J.I.; Falcó, A.; Pérez-Gracia, M.T.; Antón-Fos, G.M. opological index Nclass as a factor determining the antibacterial activity of quinolones against *Escherichia coli*. *Future Med. Chem.* **2019**, *11*(17), 2255-2262.
o. Wiener, H. Structural determination of paraffin boiling points. *J. Am. Chem. Soc.*, **1947**, *69*(1), 17-20.
p. Wiener, H. Relation of the physical properties of the isomeric alkanes to molecular structure. Surface tension, specific dispersion, and critical solution temperature in aniline. *J. Phys. Chem.*, **1948**, *52*(6), 1082-1089.
q. Randic, M.; Guo, X.; Oxley, T.; Krishnapriyan, H.; Naylor, L. Wiener matrix invariants. *J. Chem. Inf. Comput. Sci.*, **1994**, *34*(2), 361-367.
r. Niederfellner, J.; Lenoir, D.; Matuschek, G.; Rehfeldt, F.; Utschick, H.; Brügemann, R. Description of vapor pressures of polycyclic aromatic compounds by graph theoretical indices. *Quant. Struct.-Act. Relat.*, **1997**, *16*(1), 38-48.
s. Platt, J.R. Influence of neighbor bonds on additive bond properties in paraffins. *J. Chem. Phys.*, **1947**, *15*(6), 419-420.
