# Supplementary material for: Pharmacokinetic Equations Applied to Obtain New Topological Models in the Search of Antibacterial Compounds
Source: Pharmaceuticals (Basel). 2025 Jun 10;18(6):865. doi: 10.3390/ph18060865 (PMC12195795; doi:10.3390/ph18060865)
Supplement: Supplementary file 1 [file pharmaceuticals-18-00865-s001.zip › Suppl Section S2.pdf]

Suppl. Section S2: Compounds selected by the models as candidates with proven antibacterial activity.

Compound (CAS Number)	$Clas_{AB+MRT}$*	$Clas_{AB+VD}$*	$Clas_{AB+CL}$*	Ref.
Acediasulfone (80-03-5)	+	-	+	a
Acetosulfone (80-80-8)	+	-	-	a
Apalcillin (63469-19-2)	-	+	-	a
Azidocillin (17243-38-8)	+	-	+	a
Aztreonam (78110-38-0)	+	-	-	a
Balofloxacin (127294-70-6)	+	+	+	a
Carindacillin (35531-88-5)	-	+	-	a
Cefaclor (53994-73-3)	+	+	+	a
Cefdinir (91832-40-5)	-	+	-	a
Cefepime (88040-23-7)	-	+	-	a
Cefetamet (65052-63-3)	+	+	-	a
Cefixime (79350-37-1)	-	+	-	a
Cefoselis (122841-10-5)	-	+	-	a
Cefotaxime (63527-52-6)	-	+	-	a
Cefoxitin (35607-66-0)	+	+	-	a
Cefroxadine (51762-05-1)	+	-	+	a
Ceftibuten (97519-39-6)	-	+	-	a
Cephalexin (15686-71-2)	+	-	+	a
Clinafloxacin (105956-97-6)	+	+	+	a
Clometocillin (1926-49-4)	+	-	-	a
Cloxacillin (61-72-3)	+	-	-	a
Fenbenicillin (1926-48-3)	+	-	-	a
Fleroxacin (79660-72-3)	+	+	+	a
Furosemide (54-31-9)	+	+	-	b
Gatifloxacin (112811-59-3)	+	+	+	a
Gemifloxacin (175463-14-6)	+	-	-	a
Grepafloxacin (119914-60-2)	+	+	+	a
Lomefloxacin (98079-51-7)	+	+	-	a
Loracarbef (76470-66-1)	+	+	+	a
Metampicillin (6489-97-0)	+	-	+	a
Methicillin (61-32-5)	-	+	+	a
Moxifloxacin (151096-09-2)	+	+	+	a
Negamycin (33404-78-3)	-	-	+	a
Oxacillin (66-79-5)	+	+	-	a
Papuamine (112455-84-2)	-	-	+	a
Penamecillin (983-85-7)	+	-	-	a
Phenamidine (101-62-2)	-	-	+	c
Prenylamine (390-64-7)	-	-	+	d
Primaquine (90-34-6)	+	-	-	e
Propamidine (104-32-5)	+	-	-	a
Stilbamidine (122-06-5)	+	-	+	f
Temafloxacin (108319-06-8)	-	+	+	a
Tetroxoprim (53808-87-0)	-	-	+	a
Tigemonam (102507-71-1)	+	-	-	a
Tolonium (56109-24-1)	+	-	-	g

Tosufloxacin (100490-36-6)	-	-	+	a
Triforine (26644-46-2)	-	-	+	f
Wildfire toxin (40957-90-2)	+	-	+	h

*The compounds are classified as active if all three functions comprised in the model are (+) or inactive (-) if at least one of them is (-).

a. The Merck Index, 13th ed.; Merck & Co. Inc.: New Jersey, **2001**.
b. Harras, M.F.; Sabour, R.; Farghaly, T.A.; Ibrahim, M.H. Drug repurposing approach in developing new furosemide analogs as antimicrobial candidates and anti-PBP: design, synthesis, and molecular docking. *Bioorg. Chem.* **2023**, *137*, 106585.
c. Wien, R.; Harrison, J.; Freeman, W.A. Diamidines as antibacterial compounds. *Brit. J. Pharmacol.* **1948**, *3*, 211-218.
d. Szatlóczky, E. Cause, diagnosis, and chemotherapy of lactose intolerance. *Br. Med. J.* **1982**, *284*, 1405.
e. Wasi, N.; Singh, H.B. In vitro evaluation of bacteriostatic activity of metal complexes of amodiaquine and primaquine. *Inorg. Chim. Acta* **1988**, *151*, 287-289.
f. Bichowsky-Slomnitzki, L. The effect of aromatic diamidines on bacterial growth. *J. Bacteriol.* **1948**, *55*, 27-31.
g. Piotrowski, W.; Sas-Piotrowska, B.; Slizak, W. Response of *Rhizobacterium leguminosarum* (strain B-73B) to some fungicides and antibiotics. *Prog. Plant Prot.* **1999**, *39*, 845-847.
h. Kamath, A.; Ojima, I. Advances in the chemistry of β-lactam and its medicinal applications. *Tetrahedron* **2012**, *68*, 10640-10664.
