# Supplementary material for: Pharmacokinetic Equations Applied to Obtain New Topological Models in the Search of Antibacterial Compounds
Source: Pharmaceuticals (Basel). 2025 Jun 10;18(6):865. doi: 10.3390/ph18060865 (PMC12195795; doi:10.3390/ph18060865)
Supplement: Supplementary file 1 [file pharmaceuticals-18-00865-s001.zip › Suppl Section S4.pdf]

Suppl. Section S4: Compounds selected by the models as candidates without proven antibacterial activity.

Compound (CAS Number)	$Clas_{AB+MRT}$*	$Clas_{AB+VD}$*	$Clas_{AB+CL}$*
Acecarbromal (77-66-7)	+	-	-
Acrinathrin (101007-06-1)	-	+	-
Anastrozole (120511-73-1)	-	+	-
Azure A (531-53-3)	+	-	-
Azure C (531-57-7)	+	-	-
Benzathine (140-28-3)	-	-	+
Bifenthrin (82657-04-3)	+	-	-
Bopindolol (62658-63-3)	+	-	+
Bromopride (4093-35-0)	-	-	+
Butethamine (2090-89-3)	+	-	+
Capecitabine (154361-50-9)	+	-	-
Capuride (5579-13-5)	+	-	-
Cartap (15263-53-3)	+	-	-
Cyhalothrin (68085-85-8)	+	-	-
Cystathionine (535-34-2)	+	-	-
Diopterin (6807-82-5)	-	+	-
Droprenilamine (57653-27-7)	-	-	+
Enviroxime (72301-79-2)	+	-	-
Epoxiconazole (133855-98-8)	-	+	-
Ethyl biscoumacetate (548-00-5)	+	-	-
Fenbuconazole (114369-43-6)	-	+	+
Guanabenz (5051-62-7)	-	+	-
Guanfacine (29110-47-2)	-	+	-
Halofenozide (112226-61-6)	+	+	+
Imazamox (114311-32-9)	-	+	-
Indecainide (74517-78-5)	+	-	-
Isofenphos (25311-71-1)	+	-	-
Lanthionine (3183-08-2)	+	-	-
Letrozole (112809-51-5)	+	-	-
Meconic acid (497-59-6)	-	+	-
Metoclopramide (364-62-5)	-	-	+
Muzolimine (55294-15-0)	-	+	-
Myclobutanil (88671-89-0)	+	+	-
Nafamostat (81525-10-2)	+	-	-
Niceritrol (5868-05-3)	-	+	-
Octamoxin (4684-87-1)	+	-	-
Oxadiargyl (39807-15-3)	-	-	+
Pinacidil (60560-33-0)	+	-	-
Pramiverin (14334-40-8)	+	-	-
Prucalopride (179474-81-8)	+	-	-
Quinocide (525-61-1)	+	-	-
Remacemide (128298-28-2)	+	-	-
Rizatriptan (144034-80-0)	-	-	+
Sobuzoxane (98631-95-9)	-	+	-
Teflubenzuron (83121-18-0)	-	-	+

Tefluthrin (79538-32-2)	+	-	-
Terodiline (15793-40-5)	+	-	-
Toluylene blue (97-26-7)	-	-	+
Triadimefon (43121-43-3)	-	+	-
Triazophos (24017-47-8)	-	-	+
Tritoqualine (14504-73-5)	-	+	-
Tybamate (4268-36-4)	-	-	+
Yellow OB (131-79-3)	+	-	-

*The compounds are classified as active if all three functions comprised in the model are (+) or inactive (-) if at least one of them is (-).

a. The Merck Index, 13th ed.; Merck & Co. Inc.: New Jersey, **2001**.
b. Hall, L. H. MOLCONN-Z software; Eastern Nazarene College: Quincy (Massachusetts), **1995**.
c. Kier, L.B.; Hall, L.H. General definition of valence delta-values for molecular connectivity. *J. Pharm. Sci.*, **1983**, *72*(10), 1170-1173.
d. Gálvez, J.; García-Domenech, R.; Salabert, M.T.; Soler, R. Charge indexes. New topological descriptors. *J. Chem. Inf. Comput. Sci.*, **1994**, *34(3)*, 520-525.
e. Kier, L.B.; Hall, L.H. The E-state as an extended free valence. *J. Chem. Inf. Comput. Sci.*, **1997**, *37*(3), 548-552.
f. Basak, S.C.; Mills, D. Quantitative structure-property relationships (QSPRs) for the estimation of vapor pressure: a hierarchical approach using mathematical structural descriptors. *J. Chem. Inf. Comput. Sci.*, **2001**, *41*(3), 692-701.
g. Shannon, C.E.; Weaver, W. *The Mathematical Theory of Communication*; University of Illinois Press: Urbana, **1949**.
h. Moliner, R.; Garcia, F.; Galvez, J.; Garcia-Domenech, R.; Serrano, C. Nuevos índices topológicos en conectividad molecular. Su aplicación a algunas propiedades fisicoquímicas de un grupo de hidrocarburos alifáticos. *An. Real Acad. Farm.*, **1991**, *57*, 287-298.
i. Cummins, D.J.; Andrews, C.W.; Bentley, J.A.; Cory, M. Molecular diversity in chemical databases: comparison of medicinal chemistry knowledge bases and databases of commercially available compounds. *J. Chem. Inf. Comput. Sci.*, **1996**, *36*(4), 750-763.
j. Bonchev, D.; Trinajstic, N. On topological characterization of molecular branching. *Int. J. Quantum Chem.*, **1978**, *14*(S12), 293-303.
k. Kier, L.B. A shape index from molecular graphs. *Quant. Struct.-Act. Relat.*, **1985**, *4*(3), 109-116.
l. Kier, L.B. Distinguishing atom differences in a molecular graph shape index. *Quant. Struct.-Act. Relat.*, **1986**, *5*(1), 7-12.
m. Kier, L.B. An index of molecular flexibility from Kappa shape attributes. *Quant. Struct.-Act. Relat.*, **1989**, *8*(3), 221-224.
n. Suay-García, B.; Alemán-López, P.; Bueso-Bordils, J.I.; Falcó, A.; Pérez-Gracia, M.T.; Antón-Fos, G.M. opological index Nclass as a factor determining the antibacterial activity of quinolones against *Escherichia coli*. *Future Med. Chem.* **2019**, *11*(17), 2255-2262.
o. Wiener, H. Structural determination of paraffin boiling points. *J. Am. Chem. Soc.*, **1947**, *69*(1), 17-20.
p. Wiener, H. Relation of the physical properties of the isomeric alkanes to molecular structure. Surface tension, specific dispersion, and critical solution temperature in aniline. *J. Phys. Chem.*, **1948**, *52*(6), 1082-1089.

q. Randic, M.; Guo, X.; Oxley, T.; Krishnapriyan, H.; Naylor, L. Wiener matrix invariants. *J. Chem. Inf. Comput. Sci.*, **1994**, *34*(2), 361-367.
r. Niederfellner, J.; Lenoir, D.; Matuschek, G.; Rehfeldt, F.; Utschick, H.; Brügemann, R. Description of vapor pressures of polycyclic aromatic compounds by graph theoretical indices. *Quant. Struct.-Act. Relat.*, **1997**, *16*(1), 38-48.
s. Platt, J.R. Influence of neighbor bonds on additive bond properties in paraffins. *J. Chem. Phys.*, **1947**, *15*(6), 419-420.
